# Thermoplastic Post-Consumer Recyclates for Buried Infrastructure: Barriers, Classification Gaps, and a Path Toward Quality-Assured Use

**DOI:** 10.3390/polym18141702

**Published:** 2026-07-10

**Authors:** Anneke Scholz, Ricky Selle, Michael Großhauser

**Affiliations:** 1 Selle Consult GmbH, Shakespearestraße 52, D-04107 Leipzig, Germany; r.selle@selle-consult.de; 2 Fraunhofer Institute for Structural Durability and System Reliability LBF, Schlossgartenstraße 6, D-64289 Darmstadt, Germany; michael.grosshauser@lbf.fraunhofer.de

**Keywords:** post-consumer recyclate, recycled polyolefins, non-pressure pipe systems, buried infrastructure, material classification, durability assessment, long-term properties, accelerated aging

## Abstract

With the Green Deal and the Circular Economy Action Plan, the European Union aims to replace half of the fossil-based raw materials in plastics with sustainable alternatives by 2030. In the plastic pipe industry, the use of post-consumer recyclates (PCRs) remains very limited due to reduced material quality, economic hurdles, limited availability, non-specific classification requirements, and a lack of testing standards. This paper presents two interconnected contributions to the quality-assured use of PCR for buried utility infrastructure made from Polyethylene (PE), Polypropylene (PP), and unplasticized Polyvinyl Chloride (PVC-U). First, a methodological framework, based on EN 13476, defines suitability as the intersection of material classification and application-specific requirements profile. A review of the current regulatory and technical situation reveals a systemic discrepancy: requirements profiles are oriented toward virgin material, while the scope of classification for PCR remains insufficiently defined. As a result, PCR is either used without adequate suitability assessment or blended with fillers, thereby limiting recyclability. Secondly, the review focuses on additive strategies, including restabilization, compatibilization, chain modification, and recyclate-compatible functional additives. These strategies are among the main technical solutions for closing the gap between PCR properties and the product’s requirements, reducing filler dependency, and enabling the long-term use of PCR in safety-critical applications.

## 1. Introduction

Annual plastic production in the European Union now stands at just under 55 million tons, yet only slightly more than 15% of this amount comes from the circular economy [1]. This includes 14.5% mechanically recycled plastics, 1.1% bio-based plastics, and 0.2% chemically recycled plastics [1].

**Figure 1:**
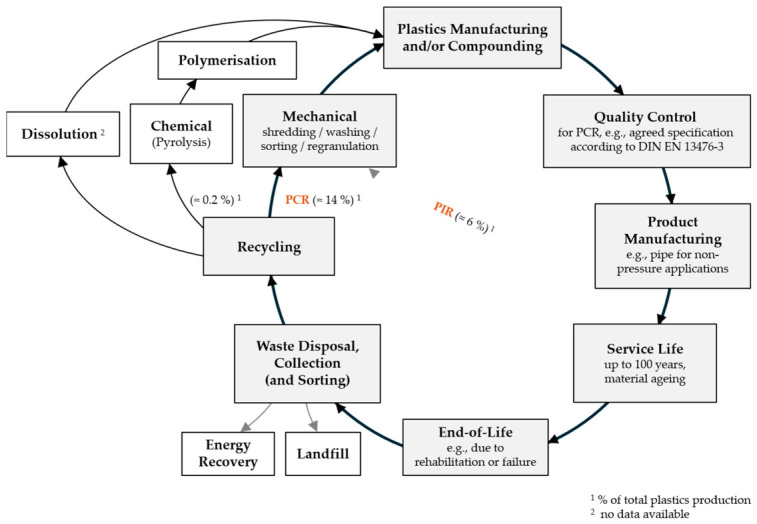
Lifecycle and recovery routes for thermoplastics, from production through use, end-of-life, and reintegration as PCR or PIR. Own illustration, based on data from [1,5].

These shares are linked to different recovery routes. Mechanical recycling remelts and reshapes the polymer without substantially altering its molecular structure and is currently the principal route for thermoplastics [2]. Chemical recycling instead depolymerizes the macromolecules via solvolysis, pyrolysis, gasification, or enzymatic routes [3]. It therefore extends the recovery to waste not suitable for mechanical recycling. However, it remains far less established, constrained by high energy demand and fragmented regulatory frameworks [2,4]. Energy recovery ranks lowest in the waste hierarchy and is increasingly disfavored due to environmental concerns [2]. For buried infrastructure addressed here, mechanical recycling is the relevant route. Figure 1 provides an overview of the plastics lifecycle and recovery routes, from polymer production through use and end-of-life collection to reintegration as post-consumer recyclate (PCR) or post-industrial recyclate (PIR).

A key approach to increasing the share from the circular economy is the increased use of so-called recyclates, defined by ISO 15270 [6] as “plastic material obtained from the recycling of plastic waste.” This refers to both post-industrial recyclates (PIRs) and an in-house production regrind, as well as post-consumer recyclates (PCRs) [7].

PIR generally offers greater consistency than PCR and often consists of a single grade, though in practice it may also contain compatible mixed fractions and be printed, pigmented, or slightly degraded [8]. It can therefore serve as an alternative when PCR is unavailable, but its use carries the risk of greenwashing, as a clear distinction from PCR is not always evident [9].

According to ISO 472 [10], PCR materials are those that originate from end users and have already served their intended purpose or are no longer usable. They are characterized by greater heterogeneity, higher levels of contamination, and more complex sorting and processing procedures [11]. Despite their inferior properties, they make a crucial contribution to the circular economy, as they enable the material cycle to be closed at the end of a product’s life and thus support resource-efficient use [12]. The use of PCR reduces reliance on fossil fuels such as crude oil and natural gas. At the same time, the use of PCR in the production of plastic products significantly reduces CO_2_ emissions [4].

As part of the European Green Deal and the Action Plan for the Circular Economy, the European Union has set clear goals: by 2030, half of the fossil-based raw materials used are to be replaced by sustainable alternatives, such as recycled materials; a complete transition is planned by 2050 [13]. These developments at the European level have a direct impact on national legislation. In Germany, for example, the Circular Economy Act applies. Under § 45(2), it requires public contracting authorities of the federal government and its subordinate agencies to give preference to products that meet ecological criteria, for example, by using recycled materials. In addition, the Federal Climate Protection Act (§ 3(2)) mandates net greenhouse gas neutrality by 2045, a goal that is increasingly putting pressure on the thermal recovery of fossil-based plastic waste. However, there is currently a significant gap between these ambitious goals and the current state of implementation.

**Figure 2:**
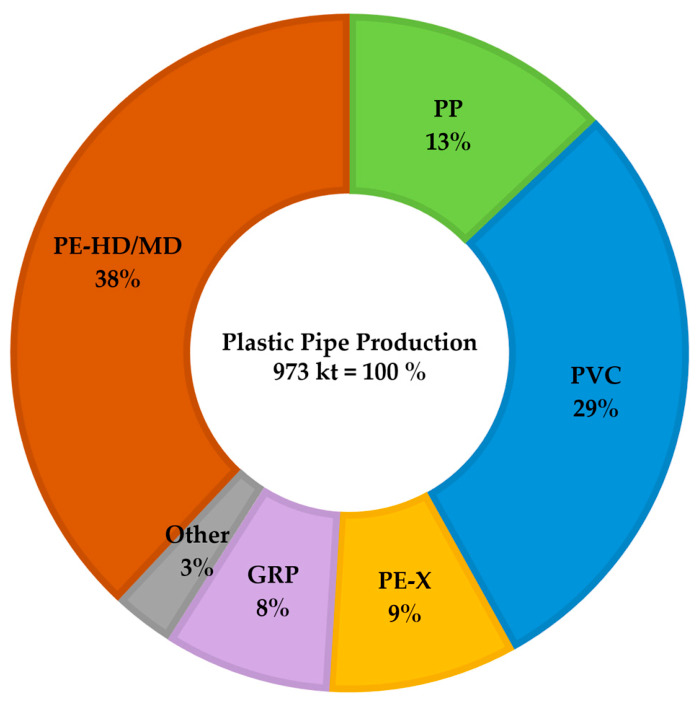
Processing volumes of plastic pipes by polymer type in Germany (2021), percentage shares derived from absolute values [16].

The use of recycled materials is currently limited in the plastic pipe industry as well [14,15]. Yet there is significant potential here: With an annual consumption of approximately 10 million tons, the European construction sector is among the largest consumers of plastics [1]. In Germany, plastics consumption is dominated by packaging (35%), followed by the construction sector (24%); the remaining 41% is distributed across vehicles (10%), electrical and electronics (6%), furniture (4%), household goods (3%), agriculture (3%), medicine (2%), and other applications (13%) [15]. In the construction sector, pipes account for 26.5% [15]. They are used in in-building piping and buried utility infrastructure, and are produced from a range of polymer types, as shown in Figure 2 [16]. Therefore, this paper focuses on this topic.

Currently, standards and technical regulations prohibit the use of recycled materials, particularly in safety- or hygiene-critical applications such as drinking water or gas supply [15,17]. Recycled materials are therefore primarily used in less sensitive applications, such as wastewater treatment, stormwater management, or cable protection [18].

In the field of standardization, there are currently relevant developments against the backdrop of the aforementioned policy guidelines, particularly in the EN 13476 series, the overarching standard for profiled plastic and multilayer pipes. For instance, Annex J was removed in the latest revision of this standard. This annex had previously explicitly excluded PCR. The requirements for mechanical material properties have also been updated. Thanks to new footnotes in the tables, the requirements regarding hydrostatic pressure test no longer apply to the middle layer of multilayer pipes, where recycled materials are primarily used. Since the middle layer typically accounts for 50–80% of the pipe cross-section according to manufacturer specifications and technical documentation, this change enables a significantly higher use of recycled materials.

Fundamentally, the reasons for the low use of PCR in the plastic pipe industry can be divided into five points, although these reasons are also representative of other industrial applications:

1. The reduced quality of PCR compared to virgin material, which manifests itself in altered processing properties and in reduced mechanical properties;
2. Economic hurdles, i.e., high prices for high-quality PCR compared to virgin material;
3. Limited availability of PCR; demand currently exceeds supply, partly because many production facilities are currently ceasing operations;
4. Requirements for classification in the regulations are too vague, such as the lack of mandatory information on relevant mechanical properties on the data sheet, i.e., no suitable classification;
5. Lack of testing standards for type testing that would enable a systematic assessment of material quality.

The lower quality of recycled materials compared to virgin materials is an inherent characteristic, regardless of the specific form. This characteristic is related to the degradation of polymers under certain conditions. Two basic forms can be distinguished: thermomechanical degradation during reprocessing and degradation during the preceding phase of use [19]. During the recycling process, the material is subjected to stresses that lead to chain scission [20]. This process has a material-dependent effect on key mechanical properties, including tensile strength, impact strength, elasticity, and viscosity [20]. The subsequent aging process during use leads to a continuous decline in initial performance levels in each cycle [20].

The higher price of high-quality PCR compared to virgin material is due, in part, to the low prices of fossil-based raw materials. This coupling of virgin polymer prices to fossil feedstock prices is volatile. Recent market disruptions have repeatedly and rapidly shifted the relative competitiveness of virgin and recycled polymers [21]. Furthermore, PCRs are, by their very nature, a mixture of materials. Although the main fractions can usually be separated by polymer type, such as PE, PP, and PVC, they are not entirely homogeneous and require extensive sorting, cleaning, and processing. The associated additional technical effort and the assessment of material quality increase production costs [4,13,15].

The limited availability of recycled materials poses a massive problem. The demand for PCR in Europe (EU-27+3) significantly exceeds the available supply. Based on the total demand for plastic pipes [22] and the Green Deal target of 50% recycled content by 2030, the calculated PCR demand is approximately 2.2 Mt.

**Figure 3:**
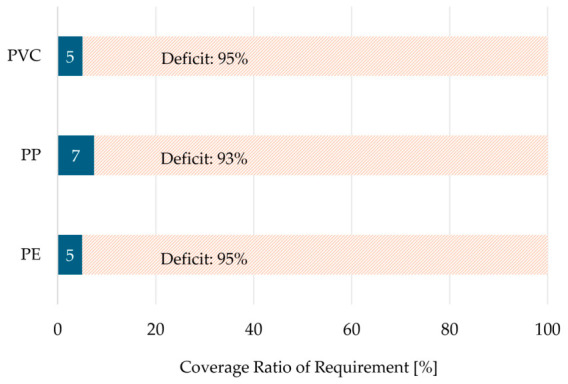
Estimated PCR coverage ratio and deficit in the plastic pipe industry in the EU-27+3 for PE, PP, and PVC calculated from EU-wide PCR production and pipe market demand data [15,16,22,23,24], and the EU Green Deal’s 50% PCR target.

To assess how much of this demand can currently be met, the available PCR volume for plastic pipes (PE, PP, PVC) was estimated in two steps: first, EU-wide PCR production figures for each material were taken from current industry reports [23,24]. Second, these were multiplied by the proportion of PCR allocated to the construction sector and, within that, the proportion used specifically for pipes [15,16]. This reveals a dramatic shortfall (Figure 3): the calculation yields an available volume of approximately 0.12 Mt, compared to a demand of 2.2 Mt. This means that currently only about 6% of the required PCR material for pipes is available if the Green Deal’s requirements were to be implemented immediately. All three materials are thus far below the Green Deal’s 50% target. The underlying data stem from the most recently published reports available at the time of writing (reference years 2023–2024) and represent a snapshot rather than a stable long-term value, given the ongoing volatility of the European recycling market.

At the same time, the industry is warning of a massive decline in European recycling capacity. According to a joint statement by Plastics Recyclers Europe, the increasing import of low-cost recycled materials, the declining demand for recycled materials produced in the EU, growing economic pressure, and high bureaucratic burdens are forcing more companies to cease operations [25]. As a result, additional recycling facilities were shut down by the end of 2025. Since 2023, this has corresponded to a decline of approximately one million tons in recycling capacity, roughly equivalent to France’s total recycling volume [25]. This development further exacerbates the situation.

Economic barriers and material availability are equally pressing issues and must ultimately be addressed alongside the technical questions. They are, however, beyond the scope of this paper, which focuses on the technical foundations of classification as one contribution to the broader solution.

Despite the differing material properties of PCR and virgin material, particularly concerning the relevant mechanical properties, only a few material specifications are currently mandatory on the data sheet according to the standard. For example, EN 13476-1 [18] specifies agreed specifications for certain material properties that must be coordinated between the recycled material supplier and the manufacturer of pipes and/or fittings. However, due to the limited availability of suitable recycled materials, there is little incentive to conduct extensive testing here as well. As a result, users must make their own assumptions regarding quality and validate them. It is well known that recycled materials also exhibit fluctuations in material quality within delivery batches, making this validation process time- and cost-intensive [26].

At the same time, it should be emphasized that European standardization has already made substantial contributions. This commitment is unique, particularly in an international comparison. A key result of this work is the new DIN EN 18065 [13], which is based on so-called Data Quality Levels (DQLs). This structure allows clear categorization of PCR by origin, material properties, and recycling process. The higher the DQL, the more information is required. The standard is supplemented by regulations for a European Digital Product Passport (DPP) [13]. It documents and transmits data uniformly throughout the entire value chain, covering two key areas: general information about the company and the material, as well as the material’s composition [13].

Since the material can generally still be sold regardless of the DQL achieved, especially given the limited availability mentioned earlier, there is currently insufficient incentive for recycled material manufacturers to provide more comprehensive information.

Furthermore, no suitable testing methods are currently available for products made from recycled materials, and the parameters considered relevant vary across different publications [27,28]. Consequently, universal type testing and the associated classification are not currently possible. According to CEN/TS 13476-4 [29], type testing is defined as testing conducted to demonstrate the conformity of molding compounds (polyolefins) or compositions (PVC) with the requirements of the relevant standard. Molding compounds/compositions are clearly defined homogeneous mixtures of a base polymer with additives.

In this paper, the term “classification” refers to determining the properties of specific recycled materials in the context of a molding compound, enabling them to be matched to a requirements profile. The identification of recycled materials as defined molding compounds, as is possible for virgin materials with Chemical Abstracts Service (CAS) numbers, is a separate topic due to inherent variations in composition and is not addressed here.

Against this background of unresolved classification and testing questions, recent research has made considerable progress on characterizing recycled polyolefins and their use in pipe applications, though largely at the material and compound level. Ragaert et al. [19] reviewed mechanical and chemical recycling pathways for solid plastic waste at a general technology level. Gall et al. [30] characterized the composition and structure-property relationships of commercial post-consumer PE and PP recyclates. Freudenthaler et al. studied the short- and long-term performance of pipe compounds containing PE [14] and PP recyclates [31] from packaging waste, with a focus on slow and fatigue crack growth resistance. Juan et al. [32] demonstrated the incorporation of recycled HDPE into PE pipe-grade resins as a closed-loop recycling route. Istrate et al. [33] assessed the environmental performance of the same route through life-cycle assessment. Messiha et al. [34] linked material-level SCG resistance to product-level pipe lifetime for PP recyclate compounds and noted that current standards are limited to fail/pass judgments.

1. Together, these studies demonstrate that the use of PCR in non-pressure pipe applications is technically feasible, although long-term performance remains a critical issue. So far, however, the systematic linking of recyclate characterization to the product-side requirements profiles defined in the relevant standards has not been addressed. The present work addresses this gap by making two contributions: A methodological framework based on EN 13476-4 [29], defining suitability as the intersection of material classification and application-specific requirements profiles. The framework places particular emphasis on long-term behavior as the core challenge of PCR classification, addressing material degradation through multiple recycling cycles, thermodynamically incompatible PE/PP blends, non-polymeric contamination, and the validity of established extrapolation methods and safety factors for PCR.
2. A review of additive strategies like restabilization, compatibilization, chain modification, and recyclate-compatible functional additives as one of the main technical solutions for closing the gap between PCR properties and application requirements without resorting to fillers that compromise recyclability.

## 2. Prerequisites for the Sensible Use of PCR

The framework proposed in this paper rests on two prerequisites that must be clarified before any increase in PCR content can be pursued: which recycled materials are available, and which applications they are suited for. This chapter addresses the question by introducing the concept of suitability as the central organizing principle.

**Figure 4:**
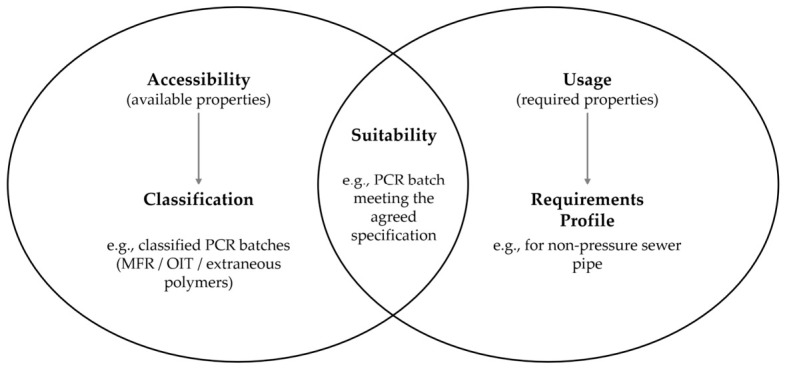
Authors’ conceptual framework illustrating the relationship between Accessibility (Classification), Suitability, and Usage (Requirements Profile).

The central prerequisite for the sensible use of recycled materials is the identification of suitable and available materials and of corresponding target products. Suitability can be described as the intersection of the classifications of various recycled materials that meet the requirements profile of specific construction products. The relationship is illustrated in Figure 4.

The required properties of the recycled materials, or requirements profiles, are determined by two factors: the mechanical and physical properties the product must possess for its intended use, and parameters related to the material’s processability in the production process. Together, these two aspects define quality. A classification must account for properties related to these two aspects, such as the melt flow rate (MFR) as a manufacturing parameter and tensile properties for the product’s structural integrity. The greatest differences compared to virgin material lie in the long-term properties and in the dependence on the number of recycling cycles (see Section 3.3.1). To determine suitability, the requirements must be cross-checked against the “existing properties” listed on the data sheet in accordance with the agreed specification.

This conceptual link between classification, suitability, and product specification provides the foundation for the method described in the following chapter, which shows a concrete sequence of quality assurance steps.

## 3. Establishment of a Quality-Assured Material Stream

### 3.1. Basic Procedure

**Figure 5:**
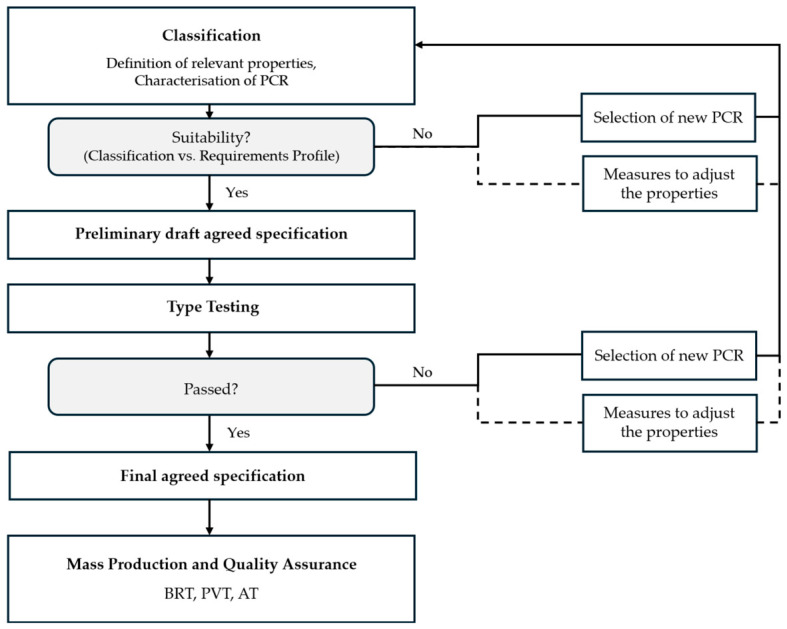
Author’s flowchart for a quality-assured material stream. Solid arrows indicate the standard process; dashed arrows indicate feedback loops triggered by a negative classification or type-testing, restarting the process at the classification stage.

After selecting a suitable recycled material based on classification and the requirements profile, a quality-assured material stream must be established; the process is illustrated in Figure 5. Approaches to technical implementation are discussed below based on the aforementioned EN 13476 [29].

The process for establishing a PCR begins with classification, during which the PCR is characterized and tested for basic suitability. In this process, relevant material properties must be defined based on the intended application; these properties are central to processability (testing of the raw material) as well as to mechanical properties (testing of the processed material). This step depends directly on the stresses acting on the material (see Section 3.2). This may sound trivial at first, but it is not, as it concerns properties after aging.

Plastic aging refers to irreversible chemical and physical changes that occur over time and can impair a material’s serviceability. These changes affect the molecular, supramolecular, or phase structure and can occur during manufacturing, processing, storage, or use [35]. Since recycled materials differ significantly from virgin materials, particularly concerning their long-term properties, it is necessary to use suitable artificial aging methods to verify the previously defined properties (see Section 3.3). Currently, there are no established test methods for this purpose, which is why this point represents the greatest challenge. It should be noted that, compared to virgin material, a more generous tolerance for variations is required. Furthermore, measures to adjust the properties are possible both before classification and after a negative classification result (see Section 3.4).

Based on the classification results, a preliminary draft of the agreed specification is prepared. This specification defines the requirements for recycled material as a raw material and serves as the contractual basis between the recycled material supplier and the pipe manufacturer. This approach focuses on the “recycled material,” that is, exclusively on the material level.

The classification of the material, therefore, directly influences its suitability and, consequently, its availability and potential applications. If necessary, it may be required to subsequently select new, suitable recycled materials, which would restart the process at the classification stage. This is followed by type testing at the material level, during which material properties such as MFR, density, OIT, thermal stability, aging behavior under defined conditions, and other parameters relevant to the application are tested. Type testing differs from classification in that the latter constitutes a preliminary screening for suitability assessment, whereas type testing provides legally binding quantitative verification of the defined requirements. The application of a suitable extrapolation method, which enables the prediction of material properties over a period of typically 50 to 100 years, is also part of the type testing. In doing so, it is necessary to consider appropriate safety factors. Only based on the results of this type of testing is the finalized, agreed specification defined, which establishes the tolerances and limit values.

Following successful type testing, series production of the recycled material can begin, with continuous validation taking place. To utilize established quality assurance processes, the recycled material manufacturer provides an inspection certificate 3.1 in accordance with EN 10204 [36] for each batch. This certificate documents that the delivered batch meets the properties specified in the agreed specification. However, the required tests do not necessarily have to be performed by the recycled material manufacturer itself.

Quality assurance during ongoing production is carried out via several mechanisms: Batch release tests (BRTs) must be successfully completed before a batch may be released. The results of these tests are documented in the inspection certificate 3.1 in accordance with EN 10204. In addition, process verification tests (PVTs) are conducted at specified intervals on the ongoing production, during which the values determined from the type test are cross-checked. In addition, audit tests (ATs) may be conducted on behalf of an inspection or certification body. Here, too, clear limits must be defined and a procedure established for non-compliance, with the process potentially needing to start over at the classification stage.

At the end of this process, a quality-assured PCR material stream demonstrates compliance with the agreed specifications through documented inspection certificates.

However, reality shows that standards for characterizing recycled materials, such as PE (EN 15344 [37]) or PP (EN 15345 [38]), do not cover this range of requirements and thus cannot demonstrate suitability for specific applications. One example is recycled PP and its potential use in multilayer sewer pipes. According to EN 15345 [38], data sheets for PP recyclates must only specify the following properties: product designation, color determined by visual inspection, form (e.g., pellets or flakes), density, melt flow rate (MFR), and, for extrusion, the melt filtration mesh size.

In contrast, EN 13476-3 [39], Annex D, requires a significantly broader agreed specification for the use of PP recyclates in multilayer pipes, i.e., an agreement between the supplier and the manufacturer that, in addition to most of the properties mentioned above, also includes the following parameters: thermal stability (OIT), ash residue, content of foreign polymers, impurities, type of pigments and/or additives, volatile substances, tensile properties, and the origin of the material. Furthermore, a long-term property is also addressed here: resistance to slow crack growth is currently a voluntary test but is intended to become mandatory in the future.

This discrepancy reflects a systemic problem: the requirements profile for and the classification of PCR are currently not aligned. While the requirements for construction products tend to be set too high or are geared toward virgin materials or PIR, the required scope of classification for PCR is not sufficiently defined. The current situation with buried utility infrastructure, therefore, presents a mixed picture: while recycled materials are already used in various construction products, they are sometimes used in applications where their suitability is critically questioned. This is the result of an insufficient suitability analysis.

For various construction products, such as pipes or infiltration elements, the gap between the existing properties and the requirement profile for mechanical properties is closed, for example, by adding fillers, such as mineral substances like talc or synthetic materials like glass fibers. In particular, the stress-based hydrostatic pressure test proves problematic, as pipes made from recycled materials generally fail it. While the use of fillers is an effective way to meet test requirements, it is problematic from a sustainability perspective. The addition described significantly limits recyclability after subsequent use, thereby effectively ending the cycle. At the same time, however, there are currently efforts at the standards level to develop and establish a new methodology that better meets the specific requirements of recycled materials.

Furthermore, the lack of specific testing procedures for recycled materials can lead to limitations in long-term behavior going unrecognized. Beyond the mechanical and chemical properties addressed by the procedure outlined above, inadequate classification can also have ecological consequences, most notably the uncontrolled emission of nano- and microplastics. Both topics are discussed in the following section.

### 3.2. Derivation of Application-Relevant Properties from the Requirements Profile

The properties relevant to the application are determined based on the design loads to which the construction products are subjected through so-called actions. The concept of actions is fundamentally defined in Eurocode 0 (EN 1990 [40]) and encompasses all forces acting on a structural system and the resulting deformations. The requirements for the resistance of construction products result from these actions on plastic pipe systems in sewer networks (see Table 1) and their characteristics. For plastic pipes, this means minimum wall thickness and minimum mechanical property values. When determining the limit values, all factors influencing the relevant long-term properties must be taken into account; see Section 3.3.

It should be noted that in most cases, poorer mechanical properties can be compensated for by correspondingly greater wall thicknesses. The following table is limited to material properties. The requirements for pipes are often formulated as properties resulting from the combination of material and geometry, e.g., ring stiffness.

The suitability of a material depends largely on its properties after aging. A distinction must be made between internal (material-related) and external (environmental) aging factors, which can accelerate the aging process and lead to premature failure [35]. Particularly in the case of thermal or photochemical aging, it should be noted that these processes are associated with a surface-degradation process that exhibits a layer-dependent gradient. This phenomenon has also been observed in field-aged pipes [49].

For most of the actions listed in Table 1, studies, scientific literature, and established testing methods are available. One relevant property, however, is not yet covered by standard test methods: the release of microplastics. It must be addressed as an element of the requirements profile. Microplastics are divided into primary microplastics (particles intended for commercial use), microfibers from textiles, and secondary microplastics (produced when larger plastic objects break down) [50]. In the case of plastic pipes, the focus is on secondary microplastics released through abrasion or decomposition. According to conservative estimates, the amount of microplastics originating from plastic pipe systems is approximately 12 g per person per year [51].

In response to this issue, various studies have been conducted, including on aged stormwater pipes [52]. However, questions remain unanswered. For example, the study examined abrasion of the inner surface caused by the flow of sand and small stones. However, wall thickness is measured in millimeters, making changes in these dimensions difficult to detect. The focus is also on the presence of microplastics in water pipes without significant mechanical or chemical attack.

Other studies on the natural aging mechanism of buried PE pipelines [53] showed that, after 13 years of service, white particles and contaminants were observed in the surface microstructure. As aging progressed, “pits” also formed, which became more numerous and deeper with longer operating times [53].

However, there are developments in this area: The European Drinking Water Directive (EU) 2020/2184 stipulates that microplastics in tap water will be monitored for the first time once a suitable measurement method has been established. This was adopted by the European Commission in March 2024 [54]: A filter cascade is used to collect particles and fibers from water intended for human consumption, with 1000 L being analyzed. The size and shape of individual particles are determined from optical microscopy images or chemical mapping, while their composition is determined by vibrational microspectroscopy [54]. With this strategy, more reliable data on the actual content will be available after 2030.

### 3.3. Evaluation of Long-Term Properties as a Classification Feature

#### 3.3.1. Material Degradation Due to Multiple Recycling Steps

Long-term properties are a key factor in assessing PCR. Premature material failure in PCR can be influenced by various factors. A significant factor is material degradation resulting from multiple recycling steps.

Polymers are generally subject to thermal-oxidative degradation throughout their life cycle, from synthesis through storage and processing to use as a final product and, in the case of recycled materials, reprocessing [30]. These processes gradually degrade polymer properties, particularly their mechanical properties [55]. Continued material degradation also occurs during multiple recycling steps, although the specific degradation mechanisms depend on the polymer. For polyolefins, two main mechanisms can be distinguished: chain branching or cross-linking, and chain scission.

The different response of PP and PE to radical formation during processing and oxidative aging arises from a fundamental chemical asymmetry in their degradation mechanisms. In PP, radical formation at tertiary carbon atoms predominantly leads to β-scission of the main chain, resulting in chain scission, molecular weight reduction, and shortening of the longest polymer chains [56]. In contrast, PE exhibits a distinct degradation pathway that also differs between LDPE and HDPE. Although chain scission initially occurs, the resulting radicals tend to recombine, promoting the formation of short- and long-chain branches. In HDPE, this recombination can even result in a net increase in molecular weight [56,57].

These different degradation mechanisms directly influence the long-term properties of the respective polymers [56]. For PE, this is particularly relevant for pipe-grade resins, whose resistance to slow crack growth and rapid crack propagation responds non-linearly to recycled content [33].

In general, long-chain branches affect the rheological and mechanical properties, while short branches influence solid-state properties such as crystallinity [57]. For PP, this specifically means that the material’s load-bearing capacity decreases [58,59]. While the mechanical properties remain relatively stable in early recycling cycles, significant changes become apparent after multiple cycles: Stiffness often increases slightly, while toughness decreases significantly [20,60]. Another characteristic feature is an increase in crystallinity, which manifests as a higher melt flow index (MFI), a higher tensile modulus, and reduced elongation at break [12,58]. In addition to the physical changes, chemical modifications also occur. In PP, antioxidants are degraded, reducing oxidation stability (OIT), and oxidation creates new functional groups, such as ketones or carboxylic acids, which can be measured using the carbonyl index [61].

PVC, by contrast, degrades by a fundamentally different mechanism. Rather than chain scission or branching, it undergoes dehydrochlorination: the elimination of HCl produces conjugated polyene sequences in an autocatalytic process [62]. This, together with its high additive load, substantially limits its recyclability [63,64].

In general, every recycling cycle alters the material. For quality assurance, comprehensive analyses of the mechanical, rheological, and chemical properties are therefore necessary [20,65].

In practice, however, the degradation effects mentioned above are mitigated by dilution. Recycled material batches are not composed of material of a uniform age, but consist of various materials that have already undergone different numbers of recycling steps [66]. As a rule, therefore, the resulting material has not undergone a very large number of processing cycles overall. In addition, recycled and virgin materials are often blended to reduce material costs while minimizing the impact of quality degradation on part performance [66]. Studies have shown that in such blends, the material typically contains only minimal amounts of heavily degraded material that has undergone numerous processing cycles [66]. Rather, the properties are dominated by the fractions that have been processed less.

#### 3.3.2. Blends of PE and PP

A second factor that affects long-term properties is the presence of thermodynamically incompatible blends, such as PE/PP. They arise from the similar densities of both polymers, which complicate the sorting of waste streams [67,68]. PE and PP, therefore, do not form a homogeneous phase, but rather a two-phase system with clearly distinct melting points [67,68,69].

The arrangement, size, and shape of the phases (i.e., the morphology) significantly influence the resulting material properties [70]. The morphology that develops, ranging from droplets of one polymer dispersed in the other to co-continuous structures, depends strongly on the blend composition [71]. Hot stage microscopy and DSC analyses of PP-based blends illustrate this [70]: The addition of PE-LD reduces the nucleation density of PP and leads to slower crystallization with smaller spherulites, whereas PE-HD, due to its crystallization temperature being nearly identical to that of PP, acts as a nucleating agent, accelerates crystallization, and, depending on the composition, even exhibits co-crystallization and partial miscibility. In contrast, PP behaves as follows in a PE matrix [70]: in PE-LD, which crystallizes at significantly lower temperatures, PP, which crystallizes first, acts as a heterogeneous nucleating agent, raising the crystallization temperature of PE-LD. In PE-HD, however, due to the overlapping crystallization temperatures, a bimodal crystallization behavior with signs of partial miscibility is observed [42].

Unmodified PE/PP blends typically exhibit poor mechanical properties because the phases exhibit only weak adhesion [67,68]. This leads to stress concentrations at the phase boundaries and, consequently, to cracking. The weak interfaces also disrupt the deformation behavior of the blend: while the individual polyolefins all deform by shear yielding, these mechanisms break down at the poorly bonded phase boundaries, thereby reducing ductility [72]. Elongation at break and impact strength decrease significantly when PP is added to PE [67]. One study [73], for example, found PCR PE-LD with up to 7 wt.% PP to be considerably more brittle than virgin PE-LD. In certain cases, the modulus of elasticity can exceed the blend rule at high PP contents, indicating interactions between the polymer chains [74]. The behavior during slow crack growth (SCG) is particularly critical. Even small amounts of foreign polymers lead to significant losses: As little as 5% PP in PE-HD can reduce SCG resistance by up to 40%, and PE in PP-B by up to 70% [68]. The resulting voids act as defects where fracture can initiate, making the fracture paths less predictable [68,75].

A major challenge currently lies in the lack of a methodology for precisely detecting PE or PP content in recycled polyolefins [30]. The chemical identification of the dispersed phases, for example, is often difficult; spectral resolution in the ATR-FTIR imaging method is often insufficient to unambiguously identify the numerous minor components of the PCR [30]. DSC analysis also often yields inaccurate fraction calculations, as they are primarily based on empirical values [76]. For quantifying the PP content specifically, FT-IR band ratios have proven more reliable than DSC, whose melt enthalpy is prone to large errors for PP copolymers [73].

#### 3.3.3. Non-Polymeric Contaminants

Whereas blends of incompatible polymers reflect a sorting problem on the polymer level, a related challenge is contamination in the form of solid impurities, which also play a significant role. Even a 10% admixture of an unsuitable recycled material containing large non-polymeric inhomogeneities can shorten the final break time by more than 30-fold [34].

Typical types of contamination according to DIN CEN/TS 17627 [77] include aluminum foil, fibers (textiles, glass), metal pieces, paper, cardboard, and sand particles. In recycled PE/PP blends, for example, nanoscale contaminants, including catalyst residues, metal particles (titanium, iron, lead, copper), and phosphorus, have been identified [67].

Depending on their distribution and type, these particles influence mechanical properties at multiple levels: Rigid inorganic particles act as stress concentrators, accelerating crack initiation and propagation [34]. The elastic and flexural moduli often increase, while elongation at break, tensile strength, and impact strength decrease [67]. Inorganic fillers such as calcium carbonate (see Section 3.1) further exacerbate the problem.

Contaminants tend to accumulate at the interfaces between PE and PP domains, forming a physical barrier there that limits the effectiveness of adhesion promoters [67]. Furthermore, inorganic contaminants such as calcium carbonate affect density-based sorting, casting doubt on the applicability of established sorting concepts [30].

Despite melt filtration, numerous particulate inclusions have been found in commercial recycled materials [30]. To better assess the service life and reliability of recycled pipes, detailed fracture mechanics studies of such inclusion defects are required, among other things.

#### 3.3.4. Extrapolation and Safety Factors

The three factors discussed (multi-cycle material degradation, thermodynamically incompatible PE/PP blends, and contamination) together determine the long-term performance of PCR. Translating this into a service-life prediction requires two methodological steps: an artificial aging method that captures the relevant failure mechanisms, and an extrapolation procedure that bridges short laboratory testing times to service lives of 50 to 100 years. Both steps pose specific challenges in PCR.

Various approaches are used for extrapolation. A commonly used method is temperature-dependent extrapolation via the Arrhenius equation, which assumes that chemical reaction rates depend exponentially on temperature [78,79]. By testing at elevated temperatures, the degradation process can be accelerated, and the service life at operating temperature can be extrapolated. However, this approach has limitations when applied to polymers: not all degradation mechanisms follow an Arrhenius relationship, and mechanisms can change with temperature [79]. Studies [79,80] show that Arrhenius plots often exhibit characteristic curvature when competing processes with different activation energies are present. At lower temperatures, the dominant mechanism shifts from high-activation-energy processes to those with lower activation energy [79]. Since low-temperature processes dominate at longer extrapolations, linear high-temperature extrapolations yield overly optimistic service-life predictions [79]. In the case of recycled materials, the question arises whether the activation energies correspond to those of virgin materials [81].

Alternatively, or in addition, extrapolation is used to account for increased mechanical loads. In this method, components are tested under higher stresses, and the results are extrapolated to the actual service stresses [82]. This approach is particularly relevant when assessing slow crack growth (SCG) [83]. SCG is the dominant failure mechanism for both polyethylene and polypropylene in long-term applications such as pipes [83]. For recycled materials, this extrapolation is particularly challenging because material behavior often exhibits nonlinear trends. A critical point here is the so-called “knee” in the creep curves, which marks the transition from ductile fracture to brittle fracture failure [84].

Various models exist for the mathematical description of the “knee,” and the choice of model significantly influences the extrapolated service life. Bilinear approaches describe the transition as an abrupt change between two linear regions and allow for a simplified distinction between sub-critical and super-critical creep behavior [85]. Hyperbolic models [86] or sigmoidal functions [87], on the other hand, represent a gradual transition and allow the depiction of the characteristic curvature in time-stress curves observed experimentally. These modeling approaches are used for both polyethylene and polypropylene [88].

Studies show that the “knee” shifts due to recycling [12]. A shift in the transition can also be observed after thermal aging [49]. Different polyolefins behave differently in this regard: While PP remains relatively stable, PE-LD can be almost completely regenerated through restabilization, whereas PE-HD exhibits a significant and progressive decline in impact strength [49].

These differing degradation profiles and the shift in critical transition points demonstrate that extrapolation methods developed for virgin materials cannot be applied to recycled materials without prior validation. Specific investigations are required to verify the applicability of established models for recycled materials and, if necessary, to develop adapted extrapolation methods. The systematic re-evaluation of extrapolation routes used in current standardization work for non-pressure thermoplastic pipes, including their applicability to recyclate materials, is the subject of ongoing research and will be addressed in dedicated publications.

There is also a need to define appropriate safety factors. According to Eurocode, for example, partial safety factors (γ-factors) are used that cover various aspects: material strengths (γ_M_), actions (γ_F_), and geometric parameters (γ_G_). For recycled materials, adjustments to these factors may be necessary to account for greater variation in material properties and additional uncertainties in service-life calculations.

### 3.4. Measures to Adjust the Characteristics of PCR

#### 3.4.1. Additive Strategies for PCR

When the assessment in Section 3.3 shows that a PCR does not meet the requirements derived in Section 3.2, two responses are possible: rejection of the material or targeted modification of its properties through the addition of additives. This chapter discusses the second option.

The degradation mechanisms, PE/PP blends, and contaminants described in the preceding sections make it clear that recycled materials often do not achieve the properties required for long-lasting products in buried utility infrastructure without further measures. In addition to optimizing sorting, processing, and processing parameters, the targeted addition of additives, in the sense of restabilizing or “repairing” the PCR, represents a central approach to improving material performance and, in the long term, enabling the use of PCR in safety-critical applications [89].

Broadly speaking, four technical control variables can be distinguished, which are often combined in practice: restabilization against thermo-oxidative and photo-oxidative degradation; compatibilization and morphology adjustment of PE/PP blends; chain modification to adjust rheology; and the targeted use of functional additives while ensuring recyclability.

#### 3.4.2. Restabilization Through Suitable Stabilizer Packages

Due to pre-aging and previous processing cycles, recycled materials exhibit reduced levels of antioxidants and increased levels of oxidized degradation products (e.g., carbonyl groups, hydroperoxides); see Section 3.3.1. This leads to decreased oxidation stability (OIT) and accelerated thermo-oxidative aging, which can be compensated for by targeted restabilization.

In practice, combinations of primary antioxidants (sterically hindered phenols), which immediately stop radicals, and secondary antioxidants (phosphites/phosphonites), which extend protection and regenerate the primary protection, are predominantly used. The antioxidants are supplemented by process stabilizers as well as, where appropriate, UV absorbers and HALS types. Numerous studies, including a comprehensive overview of 30 years of restabilization practice [89], show that such packages can significantly improve the OIT, mechanical properties, and long-term behavior of PCR-PP and PCR-PE (alongside PVC, the two main materials for pipe systems). Multiple extrusion cycles no longer lead to a continuous decline in molecular weight and toughness but can be controlled within standard tolerances.

Many stabilizing additives have been developed for optimized performance in virgin polymers. However, since recycled materials often have different properties than virgin materials, stabilizers (and stabilizer systems) can be developed specifically for recycled materials. Studies at Fraunhofer LBF have also demonstrated the possibility of supplementing classic, petrochemically based phenol/phosphite systems with bio-based antioxidants. In Mayer et al. [90], novel stearyl esters of p-hydroxycinnamic acids (p-coumaric, ferulic, and sinapic acid) were investigated in PP. The sinapic acid-based structure achieves a process stability in melt processing that is comparable to the state-of-the-art stabilizer AO-1076 and exhibits very high radical scavenging activity in the DPPH test. As expected, the long-term OIT lags behind regenerable phenol systems, making these biogenic antioxidants particularly suitable for processing stabilization of PCR polyolefins.

In two further studies on bio-based antioxidants [91,92], structurally systematically constructed, bio-based 4-hydroxybenzoates, -cinnamates, and -phenylpropionates were investigated. By varying the ortho-substituents (hydroxy vs. methoxy groups) and the side chain (benzoate vs. cinnamate vs. phenylpropionate), it was demonstrated that

- Tri-hydroxy-substituted phenylpropionates exhibit the lowest bond dissociation energies (BDE) of the O–H bond;
- Their DPPH radical scavenging rate correlates very well with process stabilization in the microextruder;
- And that, in PP model formulations, they achieve stabilization in the melt that even surpasses that of classical phenols in some cases, at moderate usage levels.

This provides structure-property-based design rules for bio-based antioxidants that can be used in the future to stabilize PCR polyolefins with a lower petrochemical additive load.

A distinctive feature of restabilization in the context of recycling is that the incoming material typically contains residues from the original stabilizer packages, so interactions with newly added additives cannot be ruled out. OIT measurements primarily detect residual phenolic compounds but provide little insight into the specific stabilizer history [89].

It is known from model systems that the combination of different stabilizer classes can have both synergistic and antagonistic effects: for example, phenols and phosphites classically reinforce each other in stabilization, while combinations of HALS and thio-based secondary antioxidants can lead to mutual deactivation via the acidic decomposition products of the sulfur compounds [93,94].

For practical application in the pipe industry, this means: On the one hand, restabilization requires robust, preferably regenerable phenol/phosphite systems for long-term safety; on the other hand, it can be supplemented by bio-based radical scavengers in the process stage to limit degradation during extrusion and forming. In terms of standards, minimum OIT values, the carbonyl index, and, where available, indicators for hydroperoxides (or their degradation) should be incorporated into the agreed specification.

#### 3.4.3. Compatibilization of PE/PP Blends

As described in Section 3.3.2, PE/PP incompatibility results in two-phase morphologies with low interphase adhesion and associated property deficiencies (toughness, SCG resistance) [68]. A key measure of improvement is compatibilization, i.e., targeted improvement of adhesion at phase boundaries.

A compatibilizer is a substance that separates two polymers from one another or connects incompatible phases in a mixture by acting between the two polymer surfaces, facilitating interactions, and thus enabling uniform, stable miscibility as well as improved mechanical properties [95].

For the PE/PP system, block and graft copolymers containing both PE and PP segments (e.g., PE/iPP multiblock copolymers or olefin block copolymers) are the most widely studied class of compatibilizers, acting through entanglement and cocrystallization with both phases [95,96]. Studies on PE/PP-containing recyclates show that even small amounts of such compatibilizers reduce the particle size of the dispersed phase [95], mitigate stress concentrations at the phase boundaries, and thus significantly improve elongation at break, tensile impact strength, and SCG resistance [96,97]. For a comprehensive overview of nonreactive polyolefin compatibilizer design, mechanisms, and experimentally reported effects, the reader is referred to the review by Lin et al. [95].

As previously described (Section 3.3.2), even a 5% foreign polymer content can significantly reduce SCG service life [68]. In subsequent studies, suitable compatibilizers shifted this critical foreign-polymer threshold to higher levels and extended the characteristic “knee” of the creep/SCG curves to longer durations.

This opens up the possibility of using higher PCR content, provided that the PE/PP blend is either limited by appropriate pre-sorting or specifically mitigated through compatibilization. At the same time, it should be noted that compatibilizers, once added, persist in the material and cannot be ‘deactivated’ in the next life cycle; they themselves become legacy additives in the subsequent recycling stream. Their effect over multiple recycling cycles must therefore be assessed within the framework of type testing and long-term studies and documented in the Digital Product Passport.

#### 3.4.4. Chain Modification and Rheological Adjustment

As described in Section 3.3.1, repeated processing of PP and PE leads to changes in the molecular weight distribution due to chain scission and/or chain branching [56]. In practice, this manifests as increased or highly fluctuating MFR values, altered melt viscosity and melt strength, and problems during extrusion and pipe production (extrudate stability, dimensional accuracy) [60,98].

In addition to restabilization, targeted chain modification represents another control parameter for adjusting a rheological window suitable for pipe production. For heavily degraded materials, chain extenders or mildly cross-linking additives are used; for high-molecular-weight fractions, controlled peroxide modification can be employed to reduce viscosity. Small amounts of organic peroxides initiate targeted homolytic cleavages that enable controlled chain scission and/or recombination, thereby temporarily increasing chain mobility.

For PE-HD recyclates, it was demonstrated at Fraunhofer LBF that the vinyl content plays a key role in degradation behavior during multiple processing [99]: PE-HD grades with high initial vinyl content (e.g., Phillips grades) tend to exhibit pronounced branching or partial cross-linking under thermo-mechanical stress (sharp drop in MVR, increase in zero-shear viscosity), whereas low-vinyl grades (classic Ziegler-Natta PE-HD) tend to show chain scission and an increase in MVR. Using high-resolution 1H-NMR spectroscopy, a quantitative relationship between vinyl functionality and branching tendency was also established for recycled materials. This method enables a well-founded selection and control of chain-modification strategies (e.g., targeted use or degradation of vinyl groups) for PCR PE-HD.

Furthermore, through multiple-extrusion series and rheological studies, it was demonstrated that suitable chain modification, in combination with restabilization, enables processable MFR windows and stable melt viscosities, even for recycled PP and PE-HD grades [99]. For classification purposes, this means that MFR limits must not be considered in isolation, but rather in the context of molecular weight distribution, degree of branching, and the modification or stabilizer concepts employed.

#### 3.4.5. Recyclability as a Design Criterion for Additive Formulation

While Section 3.4.2, Section 3.4.3 and Section 3.4.4 describe specific interventions to improve defined property deficits, the design of the additive formulation as a whole addresses an overarching question: What consequences does today’s material formulation have for recyclability at the end of the life cycle?

As described in Section 3.1, deficiencies in the mechanical properties of recycled materials are often compensated for today by high filler contents (calcium carbonate, talc, glass fibers). While this allows the material to pass demanding tests (e.g., hydrostatic pressure test), it significantly impairs recyclability, as fillers are virtually impossible to remove in subsequent recycling cycles and hinder sorting and melt filtration.

In contrast, the additive approach outlined here aims for functional upcycling: The measures described in Section 3.4.2, Section 3.4.3 and Section 3.4.4 (restabilization, compatibilization, and chain modification) are primarily intended to improve the intrinsic quality of PCR without compromising its subsequent recyclability. In this context, classic functional additives, such as carbon black for UV protection, HALS, or nucleating agents, are selected and dosed so that their effects remain targeted and their accumulation in subsequent cycles is controllable.

Current examples of such functional additives include alditol-based systems combined with α-tocopherol [100]. In various PCR-PP and PCR-PE-HD systems, it has been demonstrated that

- Alditol polyols (e.g., erythritol, xylitol, mannitol) degrade hydroperoxides, address carbonyl groups via acetal formation, and complex transition metal ions;
- At the same time, α-tocopherol is regenerated from the polyols via hydrogen transfer;
- And that this bio-based combination, in multi-extrusion tests, showed a higher melt viscosity stability over all 5 cycles than conventional phosphite-based systems, in some cases with a lower phosphorus content.

The OIT values after five extrusion cycles, as well as the mechanical properties (tensile strength, elongation at break) of restabilization packages containing alditols and α-tocopherol, were higher in PCR-PP and PCR-PE-HD than those of industrial standard formulations [100]. This provides another phosphorus-reducing approach to restabilization that is advantageous from a circular-economy perspective.

From a normative perspective, it seems sensible to include, in addition to classic parameters (OIT, ash, filler content), indicators for “recyclate-compatible” additive formulation (e.g., upper limits for phosphorus and metal content, documentation of bio-based stabilizers) in specifications in the medium term.

#### 3.4.6. State of Research and Potential of Additive Formulation for the “Repair” of Recyclates

The measures described are supported by a growing body of scientific research. In addition to general overviews on additives and restabilization [19,65,89,101], numerous studies are now available that explicitly address

- The restabilization of PCR-PP and PCR-PE (classic phenol/phosphite packages, bio-based antioxidants);
- Compatibilization and morphology control in PE/PP-containing PCR;
- Chain modification and the role of vinyl groups in PCR-PE-HD [99],
- As well as with alditol- and tocopherol-based systems as alternatives to phosphites [100].

An additional methodological innovation involves the use of DPPH assays and quantum chemical BDE calculations as screening tools for new antioxidants [90,91,92]. It has been demonstrated that the radical-scavenging rate of biogenic phenols, as determined in the DPPH test, correlates very well with process stabilization in the microextruder, thereby enabling rapid preselection of additive chemical concepts for PCR materials.

Overall, several key conclusions can be drawn from this work:

1. Additive formulation is an effective lever for “repairing” recycled materials, particularly concerning thermo-oxidative stability, process stability, and SCG behavior.
2. The potential benefits are highly dependent on the material and batch; precise characterization (including vinyl content, degree of oxidation, and blend composition) is a prerequisite for targeted strategies.
3. New, often bio-based classes of stabilizers (cinnamate/phenylpropionate-based antioxidants, alditols, α-tocopherol) open up options for reducing the petrochemical additive load without compromising performance.
4. Over-addition and additive accumulation over multiple life cycles can create new risks (NIAS issues, migration, interactions); therefore, transparent documentation, appropriate test plans, and normatively defined upper limits are necessary.
5. Additive formulation does not replace the need for systematic classification and type testing, but must be embedded within these processes (DQL, DPP, type/BRT/PVT/AT classification system, see Section 3.1).

From the perspective of this paper, the use of additives thus offers a concrete, implementable solution to mitigate the conflict of objectives between material quality, availability, and application requirements: Through restabilization, compatibilization, chain modification, and functional, recyclate-compatible additives, higher PCR content can be used in pipes without unreasonably compromising safety. At the same time, the need for fillers can be reduced, and recyclability after use can be improved. A prerequisite is that the selected additive strategies are transparently documented, technically validated through testing, and reflected in standards, thereby becoming part of the agreed specification and the Digital Product Passport.

## 4. Discussion

The analysis in this work yields three central findings for the quality-assured use of PCR in buried utility infrastructure:

1. Requirements profiles are oriented toward virgin material, while the classification scope for PCR remains insufficiently defined. This discrepancy is systemic rather than incidental. It can be addressed only through aligned development on both sides: refined classification frameworks for recyclates and recyclate-aware requirements in product standards.
2. Non-pressure applications with moderate requirements for compressive strength and long-term stress resistance are suitable candidates for defined PCR proportions. A complete substitution of virgin material does not yield optimal technical performance, nor is it economically viable in most applications [75,98], whereas blends of virgin and recycled materials or the use of PCR in specific structural layers are viable alternatives.
3. The use of additives can shift the boundary of what is achievable: targeted restabilization, compatibilization, and chain modification have been shown to restore mechanical and rheological properties to within standard tolerances, provided that additive choices are documented and embedded in the classification system.

Building on these findings, the framework has direct implications for different stakeholder groups. Pipe manufacturers can use the proposed structure to specify PCR requirements through agreed specifications that go beyond the minimum information defined by current characterization standards, such as EN 15345 [38] (PP) or EN 15344 [37] (PE). Recyclate producers are provided with a clear set of properties to characterize and document, supporting transparent communication via the Digital Product Passport.

Several questions remain open. Classification provides transparency into material properties, but on its own, does not resolve the underlying challenge of fluctuating PCR quality. Closely linked is the question of cost allocation along the value chain: who bears the additional costs of material purification, contaminant removal, and extended testing?

From a research perspective, closing the gap identified in this paper requires systematic long-term testing of representative PCR compounds and the development of PCR-specific extrapolation routes, since the applicability of extrapolation methods established for virgin materials to recyclates has not yet been confirmed. From a regulatory perspective, the classification and requirements structures proposed here can only take effect if they are anchored in the relevant CEN and ISO standards. Finally, from a market perspective, using a classified PCR that meets the agreed specification will only be possible if the availability and consistency of qualifying PCR streams improve. This depends on developments in collection and sorting infrastructure, which lie outside the scope of this work. Addressing these issues, from waste collection to product certification, will establish the necessary basis to meet the circular-economy targets of the European Green Deal.

## 5. Conclusions

This paper presents two contributions to the quality-assured use of polymeric recycled materials in buried utility infrastructure. First, a methodological framework, based on the structure of EN 13476 [29], defines suitability as the intersection of material classification and application-specific requirements profile. The framework operationalizes this intersection through a sequence of classification, agreed specification, type testing, and continuous validation, and identifies the assessment of long-term properties as the core methodological challenge. Second, the paper reviews additive strategies such as restabilization, compatibilization, chain modification, and recyclate-compatible functional additives as the principal technical lever to close the gap between recyclate properties and application requirements, without resorting to fillers that compromise recyclability.

Systematic experimental validation of the proposed framework, together with its normative anchoring in relevant CEN and ISO standards, will be essential to translate these contributions into practice.

## Figures and Tables

**Table 1:** Actions, relevant properties derived therefrom for the PCR, and corresponding test standard.

Action	Description	Relevant Material Properties	Relevant Product Properties	Test Standard (Example)
Mechanical stress	Soil stresses from the ground and traffic	Modulus of elasticity, tensile strength, yield strength, elongation at break	Creep behavior, flexural strength	DIN EN ISO 9969 [41], DIN EN ISO 527-1 [42], DIN EN ISO 899 [43]
Thermal effects	Temperature cycling, elevated operating temperature	Coefficient of thermal expansion, glass transition temperature, melting temperature	Heat resistance	DIN EN ISO 75 [44], ISO 11359 [45]
Chemical exposure	Media exposure	Chemical resistance	Media resistance	DIN EN ISO 175 [46]
Abrasion	Solid particles in wastewater	Surface hardness, density, molecular weight	Abrasion resistance	DIN EN ISO 2039 [47], DIN EN 295-3 [48]

## Data Availability

No new data were created or analyzed in this study. Data sharing is not applicable to this article.

## Abbreviations

The following abbreviations are used in this manuscript:

AT	Audit Test
ATR-FTIR	Attenuated Total Reflectance Fourier Transform Infrared Spectroscopy
BDE	Bond Dissociation Energy
BRT	Batch Release Test
CAS	Chemical Abstracts Service
CEN	European Committee for Standardization
DPP	Digital Product Passport
DPPH	2,2-Diphenyl-1-picrylhydrazyl
DQL	Data Quality Level
DSC	Differential Scanning Calorimetry
EN	European Norm
HALS	Hindered Amine Light Stabilizer
ISO	International Organization for Standardization
MFI	Melt Flow Index
MFR	Melt Flow Rate
MVR	Melt Volume-flow Rate
NIAS	Non-Intentionally Added Substances
NMR	Nuclear Magnetic Resonance
OIT	Oxidation Induction Time
PCR	Post-Consumer Recyclate
PE	Polyethylene
PE-HD	High-Density Polyethylene
PE-LD	Low-Density Polyethylene
PIR	Post-Industrial Recyclate
PP	Polypropylene
PVC	Polyvinyl Chloride
PVC-U	Unplasticized Polyvinyl Chloride
PVT	Process Verification Test
SCG	Slow Crack Growth
UV	Ultraviolet
